# A multi-modal graph-based framework for Alzheimer’s disease detection

**DOI:** 10.1038/s41598-025-05966-2

**Published:** 2025-07-02

**Authors:** Najmeh Mashhadi, Razvan Marinescu

**Affiliations:** Department of Computer Science and Engineering, University of California, Santa Cruz, CA USA

**Keywords:** Graph deep learning, Multi-modal Alzheimer’s disease detection, Compositional model, Neurological disorders, Machine learning, Diseases of the nervous system

## Abstract

We propose a compositional graph-based Machine Learning (ML) framework for Alzheimer’s disease (AD) detection that constructs complex ML predictors from modular components. In our directed computational graph, datasets are represented as nodes $n_i$, and deep learning (DL) models are represented as directed edges $n_i \rightarrow n_j$, allowing us to model complex image-processing pipelines $n_1 \rightarrow n_2 \rightarrow n_3... \rightarrow n_T$ as end-to-end DL predictors. Each directed path in the graph functions as a DL predictor, supporting both forward propagation for transforming data representations, as well as backpropagation for model finetuning, saliency map computation, and input data optimization. We demonstrate our model on Alzheimer’s disease prediction, a complex problem that requires integrating multimodal data containing scans of different modalities and contrasts, genetic data and cognitive tests. We built a graph of 11 nodes (data) and 14 edges (ML models), where each model has been trained on handling a specific task (e.g. skull-stripping MRI scans, AD detection,image2image translation, ...). By using a modular and adaptive approach, our framework effectively integrates diverse data types, handles distribution shifts, and scales to arbitrary complexity, offering a practical tool that remains accurate even when modalities are missing for advancing Alzheimer’s disease diagnosis and potentially other complex medical prediction tasks.

## Introduction

The scalability of Artificial Intelligence (AI) is vital for processing large datasets across many tasks, including object recognition, segmentation, and text and image generation. This scalability allows AI to significantly impact fields such as healthcare, finance, and autonomous systems by handling vast amounts of data for accurate decision-making^1–3^. Foundation Models exemplify this scalability by learning from extensive, diverse datasets and adapting to specific tasks through fine-tuning^4,5^. However, they show limitations in handling tasks that demand deep, specialized knowledge, like medical diagnosis^6,7^. Also, their substantial computational requirements limit their performance and deployment in resource-constrained settings.

The potential of AI to adapt and generalize to new tasks highlights the importance of meta-learning^8,9^, yet faces significant challenges due to distribution shifts and the structural limitations of current DL models. While state-of-the-art DL models demonstrate remarkable generalization abilities across various problems, their performance decreases when confronted with shifts in inputs/outputs, covariates, or hidden variables^10^. These distribution shifts can result from various factors, including environmental changes (e.g., deploying a robot in New York City after training it on the streets of London^11^), hardware upgrades (e.g., hospital scanner upgrades necessitating the retraining of medical AI models^12^), or variations in the population studied (e.g., different disease risks across countries, requiring recalibrating medical diagnosis models^13^). Moreover, the monolithic architecture of many DL models restricts their capacity to model causal relationships between variables or make predictions adhering to a specific causal structure^14^.

AI algorithms can bring tremendous value to Alzheimer’s diagnosis, a complex multimodal problem^15,16^. However, many of these models cannot easily address multimodal datasets and find it difficult to scale efficiently. The accurate and effective diagnosis of AD requires integrating diverse data types, including neuroimaging, cognitive assessments, genetic tests, and biopsies from the cerebrospinal fluid, each carrying critical but distinct insights into the disease’s presence and progression. This complexity demands a flexible and modular approach capable of efficiently handling the data’s variability, addressing distribution shifts, and adapting to evolving data.

**Fig. 1:**
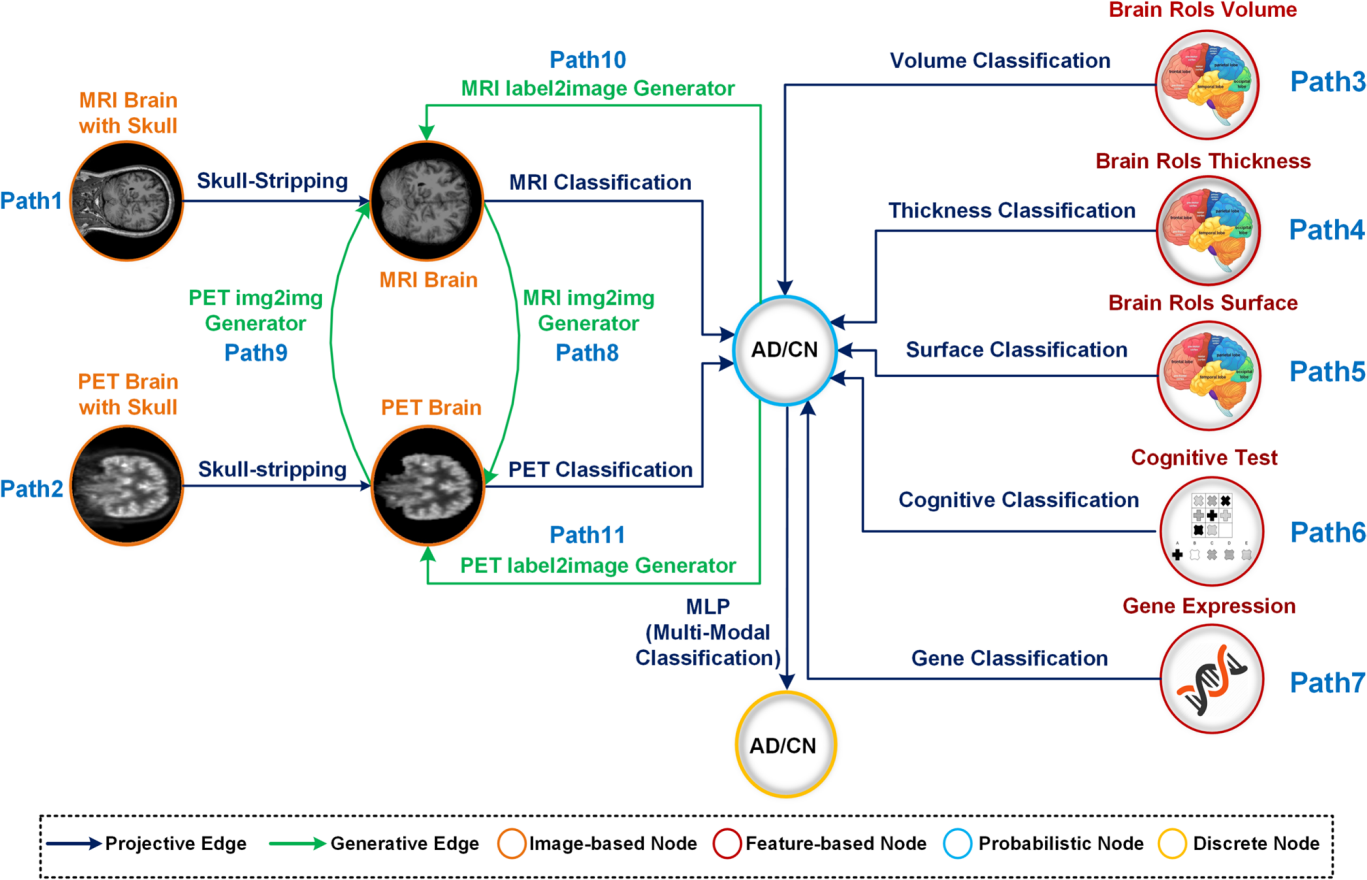
Compositional and Modular Graph for Alzheimer’s Disease Diagnosis - The proposed graph integrates various ML models and data types for AD diagnosis, where nodes represent data representations and edges denote ML models processing the data. Projective edges take data modalities and predict AD, while generative edges follow a path in the opposite direction of projective edges, branching off nodes to generate synthetic data. Classification models output probability scores for AD or CN, which are then fed into an MLP edge for the final label detection.

As illustrated in Figure 1, we propose a compositional and modular computational graph of machine learning (ML) models, A node *n* in the graph is a data representation (storing a particular dataset) and an edge $n_1 \rightarrow n_2$ is an ML model that takes input data $n_1$ and outputs data $n_2$. Any directed path in the graph becomes an ML predictor by composing the individual models. Our framework’s modularity is guided by the causal *principle of independent mechanisms* from Peters et. al.^14^, ensuring that individual components can be updated or modified without disrupting the overall functionality of the graph. We demonstrate this on Alzheimer’s Disease (AD) diagnosis, a difficult problem due to data that is multimodal, heterogeneous, and constantly evolving. Our framework integrates various data types and deep learning models to handle the complexity of this problem. For example, 2D-UNET models are employed for skull-stripping, and 2D-ResNet18 models are used for AD detection from MRI and PET scans, while Multi-Layer Perceptron (MLP) models are applied for feature-based detection, allowing the framework to analyze a broad range of data modalities. Additionally, the framework incorporates label-to-image (MRI/PET) diffusion models to generate synthetic images conditioned on diagnostic labels, augmenting the dataset to enhance detection accuracy. A StyleGAN-based generative model is used for image translation between MRI and PET scans, generating synthetic images for cases where one imaging modality is missing. This allows the model to maintain multi-modal input, capturing complementary information, and improving diagnostic accuracy, even when faced with incomplete data.

Key features of the framework include: 1) Modularity and Independence, enabling isolated updates to individual components, thus ensuring flexibility and robustness (i.e., sustained accuracy if a modality is missing); 2) Generalization to new tasks, allowing easy integration of new data types through the addition of new nodes, addressing the evolving needs of medical diagnostics; 3) Synthetic Data Generation, with generative paths enhancing detection accuracy and robustness by augmenting datasets with synthetic data; 4) Multi-modal Prediction Aggregation, where an MLP model combines classifier scores from various modalities to improve decision-making and adaptability, especially in incomplete data scenarios; 5) Differentiable Edges for Dynamic Fine-tuning, ensuring all edges are differentiable, which supports dynamic updates and fine-tuning of the graph based on feedback loss, leading to continuous improvement in precision and efficacy; and 6) Interpretability through Backward Gradient Flow, allowing the visualization of influential features at each node, enhancing the interpretability and trustworthiness of the model’s diagnostic decisions.

To train and test the deep learning models forming the edges of our graph, we employed two significant datasets: the Alzheimer’s Disease Neuroimaging Initiative (ADNI)^17^ dataset and the OASIS-3 MRI dataset^18^. The ADNI dataset served as the primary resource for training all models within the framework, offering a comprehensive collection of multi-modal data, including MRI, PET scans, cognitive assessments, and genetic information. The OASIS-3 MRI dataset was specifically used for training the MRI skull-stripping model. In all experiments, we used a 5-fold cross-validation approach to evaluate the AD/CN classification accuracy, ensuring robust evaluation and reliable performance metrics across different data splits.

## Results

### Individual performance evaluation of deep learning models at each edge

We evaluated several deep learning (DL) models that form the edges of our computational graph across different processing paths, with the results summarized in Table 1. All values are obtained across five cross-validation folds. The skull-stripping models (Paths 1 and 2) demonstrated strong performance, achieving Intersection over Union (IoU) scores exceeding 97.3%, effectively removing the skull from MRI scans.

For our image-based and feature-based AD diagnosis models (middle of Table 1), we calculated subject-based Balanced Accuracy (BAC) to account for the imbalance between Alzheimer’s Disease (AD) and cognitively normal (CN) subjects in the dataset (Supplementary Table S1). Among these models, the cognitive tests MLP model achieved the highest accuracy at 95.2% in detecting AD, outperforming other models such as the PET classifier (85.8%) and the MRI classifier (82.2%).

The bottom section of Table 1 presents the evaluation of the four generative models. The StyleGAN-based img2img generative model^19^ demonstrated superior image quality compared to the label-to-image diffusion model, as reflected by its higher Fréchet Inception Distance (FID) scores^20^. This difference likely arises from the img2img model’s capacity to better retain spatial and textural information through its direct image translation process. Supplementary Figures S1 and S2 provide visual examples of synthetic images generated by our label-to-image and image-to-image models.

**Table 1:** Evaluation of all our deep learning models (graph edges) across multiple processing paths.

Edge (DL Model)	Path	Architecture	Score
**Skull stripping**			**IoU**
MRI Skull-Stripping	1	U-Net	98.1%
PET Skull-Stripping	2	U-Net	97.3%
**AD/CN diagnosis**			**BAC**
MRI-to-Label Model	1	ResNet18	82.2±2.9
PET-to-Label Model	2	ResNet18	85.8±2.4
ROIs-Volume-to-Label Model	3	MLP	77.6±4.1
ROIs-Thickness-to-Label Model	4	MLP	66.6±1.7
ROIs-Surface-to-Label Model	5	MLP	62.2±4.8
APOE4-to-Label Model	6	MLP	58.0±7.0
Cognitive-Tests-to-Label Model	7	MLP	95.2±1.3
**Generative models**			**FID**
MRI-to-PET Model	8	StyleGAN-based img2img	68.0±3.9
PET-to-MRI Model	9	StyleGAN-based img2img	38.1±3.3
Label-to-MRI Model	10	Conditional Latent Diffusion	156.0±4.5
Label-to-PET Model	11	Conditional Latent Diffusion	140.2±6.3

### Ensemble predictions improve classification performance, weighted ensemble improves even more

We used the different paths toward the AD/CN clinical diagnosis to build an ensemble of seven methods comprising the MRI-to-AD/CN, PET-to-AD/CN, Volume-to-AD/CN, Thichness-to-AD/CN, Surface-to-AD/CN, CognitiveTests-to-AD/CN, and Gene-to-AD/CN. Results are shown in the top part of Table 2. We used ensembling through majority voting which showed a reasonable accuracy of 89.2% when all features were present; however, its performance declined to 77.2% when missing up to 5 features. The ensemble method also struggled when key features like the cognitive tests were excluded, with accuracy dropping to 85.1% (middle part of Table 2). These results highlighted the limitations of this approach in handling incomplete data.

To address the challenge of missing data, we implemented a dynamic strategy by replacing the ensemble method with a 4-layer MLP model, which learns the importance of each feature during training, enabling it to make robust predictions even when data is incomplete. A random proportion of graph classifier outputs was masked during the output MLP training to simulate varying levels of missing information.During the testing phase, we assessed the balanced accuracy of the output MLP model across varying numbers of missing features, from 0 to 5, with results averaged over 100 repetitions. The MLP model consistently outperformed the traditional ensemble method, achieving a balanced test accuracy of 94.3% when no features were missing (top part of Table 2). As the number of missing features increased, the MLP model’s performance remained relatively stable, with accuracy only dropping by 1.1% to 93.2% when five features were missing, demonstrating its robustness to missing modalities.

Additionally, excluding cognitive tests, which are highly informative for AD/CN diagnosis^21^, resulted in the highest drop in accuracy. However, the MLP model still achieved 91.3% accuracy, significantly outperforming the ensemble method’s accuracy of 85.1%, showing that using the MLP gives better performance, with minimal losses and highlighting the framework’s ability to sustain accuracy when the highly informative modality is unavailable (middle part of Table 2).

In addition to individual and randomly missing features, we also examined the effect of removing a feature subset in the bottom part of Table 2. Specifically, we tested performance when all three ROI features (surface, thickness, volume) were excluded, when both MRI and PET were removed, and when cognitive tests plus gene expression were simultaneously missing. The ensemble method displayed the largest drop when cognitive and gene features were removed (BAC decreasing to 83.5±0.04), emphasizing the pivotal role of these modalities in accurately distinguishing AD from CN. Meanwhile, the MLP model still maintained relatively high accuracy (91.2±0.03), underscoring its resilience to missing data. Also, removing surface, thickness, and volume (three ROI-based features) had a smaller impact on both methods, with the MLP dropping only about 1% from its full-modality baseline.

**Table 2:** Balanced Accuracy (BAC) of Ensemble and MLP Methods Under Random, Single, and Multi Feature (data modality) Removal Scenarios.

Impact of Missing Features on Model Accuracy (Averaged over 100 Iterations)								
Number of random missing features	0	1	2	3	4	5		
Ensemble BAC	89.2.2±0.02	88.2±0.03	87.1±0.02	87.3±0.02	82.7±0.02	77.2±0.03		
MLP BAC	94.3±0.02	94.1±0.02	93.8±0.02	93.7±0.02	93.4±0.02	93.2±0.02		

Impact of Removing Individual Features on Model Accuracy								
Missing feature	No missing feature	Cognitive Tests	Gene	MRI	PET	ROIs-Surface	ROIs-Thickness	ROIs-Volume
Ensemble BAC	89.2±0.02	85.1 ±0.04	87.7±0.05	88.2±0.05	86.6±0.05	88.9±0.03	88.8±0.02	88.9±0.02
MLP BAC	94.3±0.02	91.3±0.03	93.1±0.02	93.6±0.02	93.2±0.02	93.7±0.02	93.6±0.02	93.9±0.02

Impact of Removing Feature Subsets on Model Accuracy								
Missing feature subset	No missing feature subset	Surface + Thickness	PET + MRI	Cognitive + Gene				
Ensemble BAC	89.2±0.02	88.3±0.02	85.0±0.05	83.5±0.04				
MLP BAC	94.3±0.02	93.2±0.02	93.5±0.02	91.2±0.03				

The top sub-table reports BAC when 0–5 features are randomly omitted (averaged over 100 runs), the middle sub-table presents BAC for single feature removal, and the bottom sub-table details the impact of removing entire feature subsets. All values are mean ± standard error across five cross-validation folds. All rows use only real modalities.

### Additional external validation on AIBL and MIRIAD datasets

To further assess the generalization, we applied the ADNI-trained ensemble and MLP classifiers to two external datasets: AIBL^22^ and MIRIAD^23^. These datasets differ substantially from ADNI in terms of imaging modalities and demographic composition, which introduces domain shifts that challenge model performance. AIBL includes T1-weighted MRI, PiB-PET, and limited cognitive assessments, but lacks FDG-PET and the broader cognitive features required by our pipeline. As a result, we used only MRI images and FreeSurfer^24^- derived ROI metrics (surface area, thickness, and volume) from the MRI scans, we fine-tuned the graph components relevant to these modalities using approximately half of the AIBL dataset (53 AD, 305 CN) and evaluated the updated model on a test set of roughly equal size. MIRIAD provides only T1-weighted MRI for 69 subjects (46 AD, 23 CN), with no PET or extensive cognitive data available; for this dataset, we evaluated the unchanged ADNI-trained models without additional fine-tuning due to its limited size; the results should be interpreted cautiously given the small sample size. Table 3 summarizes the Balanced Accuracy (BAC) for each dataset. As expected, performance decreases on external datasets due to domain shifts, scanner differences, and missing modalities. However, a brief fine-tuning stage on AIBL mitigated some of these domain shifts, underscoring the importance of efficient adaptation strategies for real-world clinical applications.

**Table 3:** Balanced Accuracy (BAC) for Ensemble and MLP classifiers trained on ADNI and tested on AIBL or MIRIAD. MIRIAD lacks sufficient data for fine-tuning.

Test dataset	ADNI (7 modalities)	AIBL without finetuning MRI + Surface + Volume + Thickness	AIBL with finetuning MRI + Surface + Volume + Thickness	MIRIAD without finetuning MRI + Surface + Volume + Thickness
Ensemble BAC	89.2±0.02	82.5±0.03	84.0±0.03	80.2±0.05
MLP BAC	94.3±0.02	88.7±0.02	90.2±0.02	86.1±0.03

### Synthetic PET images improve AD prediction in cases of missing PET Data

To demonstrate the flexibility and utility of transferring data between arbitrary nodes in our graph, we explored whether finetuning the PET-to-label classifier with synthetic images generated by the MRI-to-PET translation model could enhance its performance. The balanced accuracy of the PET-to-label model improved from 85.8% ± 2.4% before finetuning to 88.4% ± 2.4% for the real test PET images after incorporating the finetuning synthetic images. This improvement might be attributed to the increased data diversity introduced by the synthetic PET images, which enhanced the model’s ability to generalize. Since significantly more subjects had MRI scans (3,788) than PET scans (1,496), the addition of synthetic PET images could have provided the model with more varied training examples, allowing it to better learn relevant patterns across modalities.

In a separate experiment, we evaluated the impact of improving the final MLP decision by incorporating synthetic PET images, simulating a scenario where test subjects lack real PET scans, a common issue in clinical settings due to the high cost and radioactivity associated with PET imaging. We generated synthetic PET images from real MRI scans using our MRI-to-PET translation model and assessed their effect on the MLP’s performance. As already reported in middle part of Table 2, removing the real PET scores lowered the MLP’s BAC from 94.3% to 93.2%. However, incorporating the classifier scores from synthetic PET raised BAC to 93.9%, showing that synthetic PET can supply complementary information and partly restore performance when real PET is unavailable.

### Skull-stripping improves AD prediction

In one of the experiments, we investigated the effect of skull-stripping on the accuracy of the MRI/PET-to-label classification models. Skull-stripping is a preprocessing step that removes non-brain tissues (such as the skull) from neuroimaging data, focusing the analysis solely on the brain regions. To evaluate the importance of this step, we compared the model’s performance with and without skull-stripping applied to the MRI and PET images, as summarized in Table 4.

Our results demonstrated a significant improvement in classification accuracy when skull-stripping was applied. Specifically, the balanced accuracy of the PET-to-label model increased by approximately 4% when skull-stripped images were used, compared to when the images retained skull features. This enhancement suggests that including skull information can introduce noise and irrelevant features that do not contribute to the detection of Alzheimer’s disease. The brain regions, rather than the skull, contain critical biomarkers for Alzheimer’s, and removing the skull ensures that the model focuses on relevant anatomical and functional characteristics.

**Table 4:** Skull-stripping effect on MRI/PET classifier accuracy.labeltab:skullspsstripping.

	MRI classifier	PET classifier
Without skull-stripping	78.2%	82.0%
With skull-stripping	82.0%	85.8%

### Incorporating the classifier loss improves synthetic image generation

To evaluate the effectiveness of the cyclical loop between the label-to-image (MRI/PET) diffusion models and the MRI/PET-to-label classifiers (illustrated by the loops between paths 1 and 10, and paths 2 and 11 in Fig. 1), we conducted two experiments. In the first experiment, the label-to-image diffusion models were trained without incorporating the MRI/PET-to-label classifier loss function for updating their weights. In the second experiment, we included the classifier loss function (Eq. 3), allowing the diffusion model weights to be updated in response to the feedback from the MRI/PET-to-label classifiers.

We then evaluated the diffusion models trained under both conditions by testing their ability to generate synthetic images that correctly represent the target class (AD or CN) while also preserving realistic textural features. This evaluation was performed using pre-trained MRI/PET-to-label classifiers to assess the fidelity and diagnostic relevance of the generated images. The results, summarized in Table 3, show that the diffusion models trained with the incorporation of the classifier loss function outperformed those trained without it. The models demonstrated an improvement in generating synthetic images that better matched the target diagnostic class.

We also repeated this experiment for the image-to-image translation models (Paths 8 and 9), integrating the loss from our pre-trained AD and CN classifier into the training of the img2img GAN generators (Eq. 2). Incorporating the classifier loss function similarly led to a significant improvement in the generated images’ ability to accurately reflect their diagnostic class as presented in Table 3. The models trained with this additional feedback produced synthetic MRI and PET images that not only retained essential anatomical and textural details but also better aligned with the diagnostic categories of AD and CN.

**Table 5:** Impact of classifier loss incorporation on synthetic image class relevance accuracy in diffusion and GAN models.abeltab:impactspsclassifierspsloss.

Missing feature	Without classifier training loss	With classifier training loss
MRI-to-label	75.3%	78.4%
PET-to-label	73.6%	77.8%
MRI-to-PET	71.2%	77.3%
PET-to-MRI	67.5%	74.7%

### Saliency map computation across the entire graph

Our graph framework is designed with differentiable edges, ensuring that any directed path from one node *X* to another *Y* is differentiable, which enables backpropagation along the entire path from *Y* to *X*. This design allows us to compute saliency maps at each node, identifying the most influential input features in the Alzheimer’s Disease (AD) detection process. To generate these saliency maps, we used the FullGrad approach^25^, a class-agnostic method that analyzes gradients across all layers of a deep learning network.

**Fig. 2:**
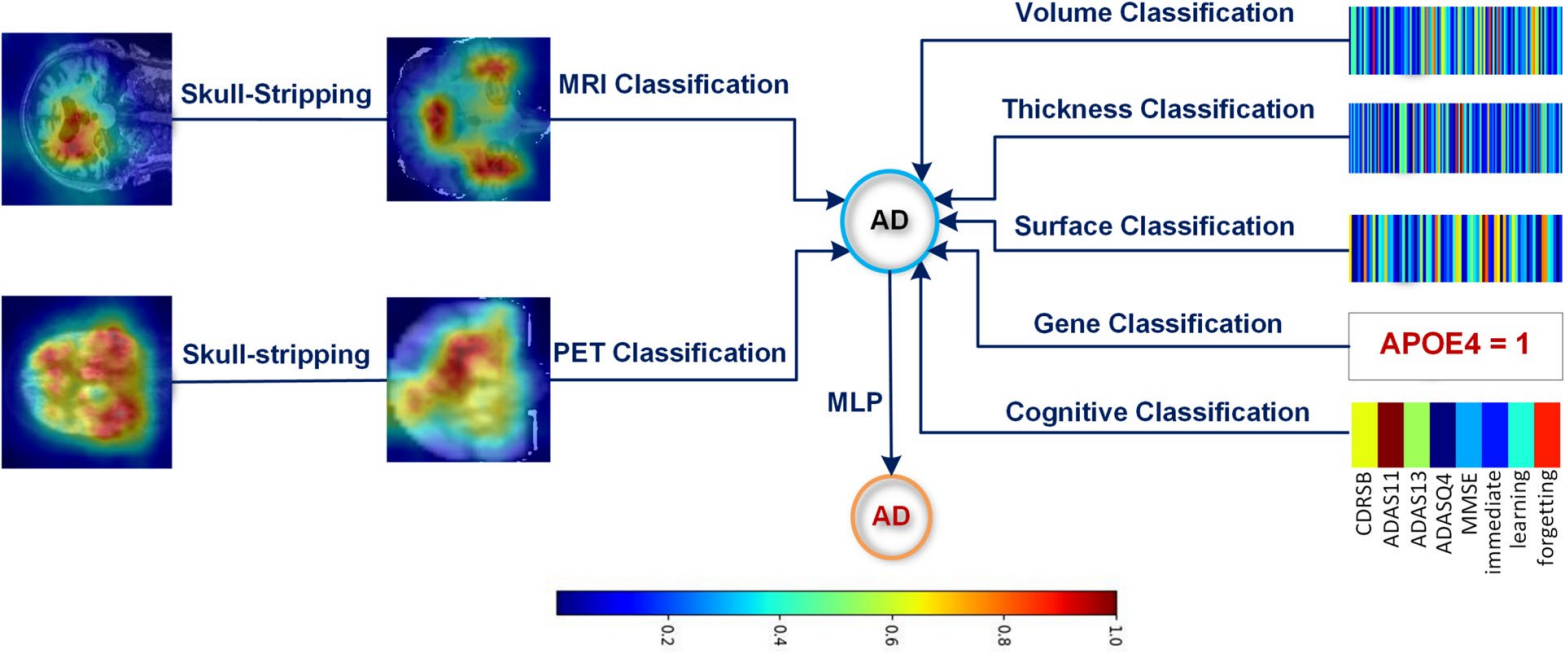
Saliency maps computed at each node in the graph for an AD subject. Red color in the saliency maps represents the most important features, and dark blue shows the least important features for AD detection.

Figure 2 presents saliency maps for an AD sample throughout our graph, providing a detailed view of the most salient features at each node.Saliency maps provide a valuable means of interpretability by revealing which features are most critical at different stages of our diagnostic pathway. In particular, they help clarify how individual modalities, MRI, PET, genetic data, or cognitive assessments, affect the final AD/CN classification. By allowing saliency map computation from any node (i.e., data representation) across paths of arbitrary length, our framework offers flexibility in exploring diverse diagnostic routes and identifying potential weaknesses or biases in the models.

However, we acknowledge that saliency-based visualization techniques have well-documented limitations. Repeated runs of the FullGrad procedure with different random seeds revealed that while most high-saliency regions consistently reappeared, some variability remained, indicating that these maps are not perfectly stable. As discussed by Borji^26^ and Zhang et al.^27^, saliency maps can be prone to artifacts such as noisy gradient estimations or biases arising from the network architecture and training data. They may not always capture causal relationships, and certain visually prominent regions in a heatmap may reflect coincidental correlations rather than genuine disease markers. Thus, while saliency maps aid in explaining our graph model’s behavior, they should be interpreted with caution, ideally in conjunction with other interpretability methods (e.g., SHAP or feature-level ablation) to cross-check the consistency of important features.

### Edge-level feature importance in multi-modal integration

**Fig. 3:**
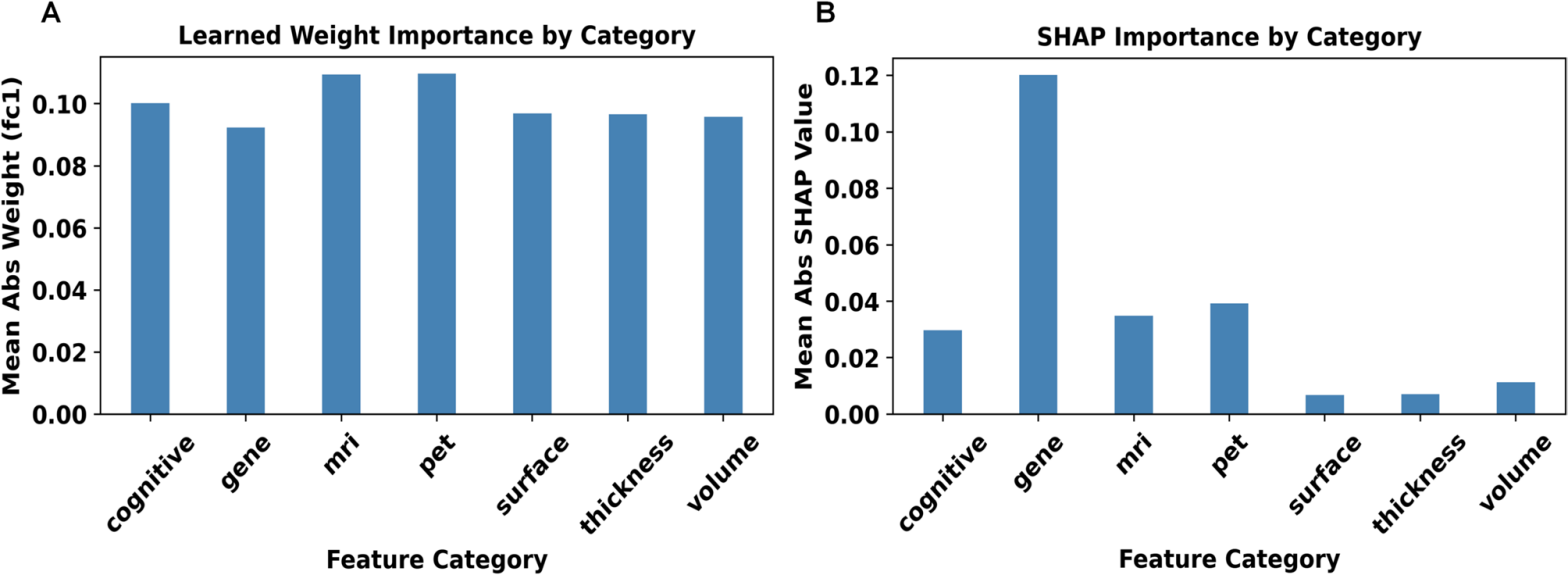
Comparison of Edge-Level Feature Importance via (**A**) Learned Weights in the First MLP Layer and (**B**) SHAP Values in the Final Model Output.

To understand how each modality shapes the final decision, we performed two complementary analyses on the output MLP, which receives AD/CN scores from every upstream sub-classifier. We first examined the absolute weights in the MLP’s initial dense layer (fc1) and averaged them by feature category (Figure 3A). These early weights are almost uniform. PET and MRI weights are slightly larger, but no modality dominates at this stage.

We then calculated SHAP values^28^ on the test set to capture each modality’s end-to-end influence (Figure 3B). SHAP measures how much a single input changes the prediction once the rest of the inputs are held constant. SHAP gives the greatest marginal importance to gene scores, followed by PET and MRI; cognitive tests rank fourth, and structural ROI metrics (volume, thickness, surface) contribute the least. Because gene score contributes information not captured by MRI, PET, or cognitive paths, SHAP assigns it the greatest influence. In contrast, MRI and PET scores are highly correlated, so once one is present, the other has only a small incremental effect on the final prediction, resulting in lower SHAP. Cognitive scores lie somewhere in the middle: they are useful but partly overlap with the imaging and gene signals. Finally, the three structural ROI metrics have significant overlap with imaging modalities so that removing any single one barely affects the output, explaining why SHAP ranks them last.

## Discussion

In this study, we introduced a multi-modal, compositional graph-based Machine Learning (ML) framework for Alzheimer’s disease detection, designed to integrate diverse data types and deep learning models in a flexible and adaptive manner. Our framework represents datasets as nodes and deep learning models as edges, allowing for the construction of complex image-processing pipelines as end-to-end ML predictors. This graph-based approach enables the combination of various data modalities, such as MRI, PET, genetic markers, and cognitive assessments, to enhance diagnostic accuracy.

Our proposed multi-modal, compositional graph framework for Alzheimer’s disease detection demonstrates promising performance, offering several paths that achieve a balanced accuracy of 94.3%—comparable to state-of-the-art methods.

Across all single- and multi-modality ablations in Table 2 BAC fell by no more than three percentage points, and five-fold cross-validation showed a variance below 0.4 percentage points. Although cognitive tests provide the highest stand-alone accuracy, they are often unavailable in clinical practice. Leveraging MRI, PET, and genetic markers allows the output MLP to maintain high accuracy when cognitive scores are missing.

Sailency map, MLP weight inspection, SHAP attribution, and ablation experiments together provide a picture of modality importance. Gene scores receive the highest SHAP values because they add information that neither imaging nor cognitive paths capture; they complement those modalities rather than replace them. Removing cognitive tests causes the largest drop in balanced accuracy, while PET and MRI are highly correlated, so once one is present, the other offers only a modest incremental gain. The three structural-ROI features (surface, thickness, volume) overlap so much that omitting any single one has a negligible effect. Also, the results confirm that the fusion MLP can still adapt and maintain good performance when some modalities are missing.

Also, synthetic data and preprocessing steps further enhanced performance. Fine-tuning the PET-to-label classifier with MRI-derived synthetic PET lifted BAC from 85.8% to 88.4%, showing that generated images can compensate when real PET is not available. Removing the skull from MRI and PET scans also improved classification, presumably because the models then focus on brain tissue rather than being distracted by irrelevant bone structures.

Besides, external validation demonstrated the models’ generalization while revealing challenges related to domain shifts. Models trained on ADNI showed lower accuracy on the AIBL and MIRIAD datasets, due to differences in imaging hardware and protocols and missing modalities; however, a lightweight fine-tuning on a small AIBL subset partially recovered the loss, underscoring the importance of efficient domain-adaptation strategies for successful real-world deployment.

Although having a framework composed of separate, reusable modules makes inference and training slightly less streamlined than a single, fully end-to-end network, each edge runs in sequence and gradients need not flow through the entire path at once, the modular graph brings practical advantages: new modalities can be plugged in with minimal code changes, existing edges can be fine-tuned or replaced without retraining the whole system, and the framework continues to operate when one or more modalities are missing.

For future work, we aim to expand the framework to incorporate additional data modalities such as different MRI contrasts (FLAIR, FSE, diffusion weighted imaging), amyloid- and tau-PET tracers, and resting-state fMRI and mild-cognitive-impairment (MCI) data; to measure how stable the saliency maps remain under adversarial perturbations and domain shifts; and to test the approach on other neuro-degenerative diseases or complex medical conditions, thereby enhancing its versatility and potential impact on medical diagnostics.

## Methods

### Graph overview

Our proposed framework, illustrated in Fig. 1, is a multimodal, compositional graph designed for Alzheimer’s disease detection. The graph comprises nodes that represent data at different stages, ranging from raw medical images to processed features and edges that correspond to deep learning models that transform and convey data between these stages.

A node *n* is a data representation with an associated space $\Omega _n$. Therefore, for a datapoint *x* to belong to a particular node *n*, it must be that $x \in \Omega _n$. An edge between nodes *n* and *m* is a differentiable transformation function $f_{nm}: \Omega _n \rightarrow \Omega _m$ that maps data from $\Omega _n$ to $\Omega _m$.

For a directed path *P* through a series of nodes $(n_1 \rightarrow n_2 \rightarrow ... \rightarrow n_k)$ with associated spaces $(\Omega _{n_1}, \Omega _{n_2}, \ldots , \Omega _{n_k})$ and differentiable transformation functions $(f_{12}, f_{23}, \ldots , f_{(k-1)k})$, the composite function $F_P$ that maps $x \in \Omega _{n_1}$ to $\Omega _{n_k}$ is:

1 $$\begin{aligned} F_P(x) = f_{(k-1)k} \circ f_{(k-2)(k-1)} \circ \cdots \circ f_{12}(x), \quad \text {where} \quad f_{i(i+1)} : \Omega _{n_i} \rightarrow \Omega _{n_{i+1}} \text { for } i = 1, 2, \ldots , k-1. \end{aligned}$$

The differentiability of each $f_{i(i+1)}$ allows for gradients of a loss function *L* to be propagated backward from $n_k$ to $n_1$ using the chain rule.

Our computational graph design is guided by several key requirements. First, all edges at the node interfaces must seamlessly integrate without requiring additional transformations. For instance, if a model’s outputs need adjustments, such as matrix transposition, normalization, or resizing to ensure compatibility with subsequent nodes, these operations should either be directly incorporated into the initial model’s outputs or handled through an additional edge in the graph. Second, the graph requires auto-differentiability along all paths. Thus, only Deep Learning models are allowed for edge creation; however, extensions to include other types of models may be considered in the future. Third, to facilitate the computation of forward and backward passes, all models must be implemented in a single deep learning framework, such as PyTorch, TensorFlow, or JAX. We chose PyTorch due to its widespread adoption and flexibility.

As shown in Fig. 1, our framework includes multiple paths, each dedicated to analyzing a specific data modality. The graph contains 11 nodes and 14 edges, forming 11 distinct paths: Path 1 for MRI to AD prediction, Path 2 for PET to AD prediction, Paths 3-5 for AD prediction using different extracted features from MRI, Path 6 for AD prediction using genetic data (specifically, the presence of the APOE $\epsilon 4$ allele), Path 7 for AD prediction using cognitive tests results, Paths 8 and 9 for generating synthetic MRI and PET images through image translation between MRI and PET scans, and Paths 10 and 11 for generating synthetic MRI and PET images based on AD/CN diagnoses.

To illustrate these concepts, consider Path 1 from Fig. 1. This path involves MRI to AD prediction and consists of three nodes: the MRI brain scan $n_1$, the skull-stripped MRI scan $n_2$ and the AD/CN clinical diagnosis probability $n_3$. The transformation functions along this path are the skull-stripping model $f_{12}$ and the brain MRI classification model $f_{23}$. Thus, the composite function for Path 1 can be expressed as $F_{P1}(x) = f_{23}(f_{12}(x))$ where $x \in \Omega _{n_1}$ is the input MRI brain scan with the skull, $f_{12}(x) \in \Omega _{n_2}$ is the skull-stripped brain MRI scan, and $f_{23}(f_{12}(x)) \in \Omega _{n_3}$ is probability score of the final classification label.

### Datasets

In our study, we employed two significant datasets for training and testing the edges of our graph: the Alzheimer’s Disease Neuroimaging Initiative (ADNI) dataset^17^ and the OASIS-3 MRI dataset^18^. ADNI was the primary dataset used for training all the models, while the OASIS-3 MRI dataset was only used for training the MRI skull striping model. ADNI is a longitudinal project focusing on brain screening and Alzheimer’s diagnosis across multiple modalities. Supplementary Table S1 lists the number of subjects analyzed, the types of data modalities available, as well as their diagnostic status (Alzheimer’s disease (AD) or cognitively normal (CN)).We addressed the inconsistencies of ADNI scans (such as varying image orientations, voxel size differences, and non-standardized metadata) using the Clinica software^29^ by converting the data into the Brain Imaging Data Structure (BIDS) format^30^ for easier access.The OASIS-3 MRI dataset comprises 1,200 MRI scans, including both raw images and skull-stripped brain images. For the test set, we selected a subset that has all seven classifier features (Fig. 1) in common, ensuring that each test subject included complete information across all modalities used in our framework (e.g., MRI, PET, cognitive assessments, and genetic markers). This approach ensured a consistent and comprehensive evaluation of the model’s performance across multiple data types and modalities.

In addition to these training and internal testing datasets, we performed external validation using the Australian Imaging, Biomarkers & Lifestyle (AIBL) study^22^ and the Minimal Interval Resonance Imaging in Alzheimer’s Disease (MIRIAD) dataset^23^. AIBL provides T1‑weighted MRI, PiB‑PET (amyloid tracer), and a limited set of cognitive assessments. We used MRI scans of 105 AD and 609 CN subjects. Because our ADNI-trained pipeline relies on FDG-PET and a broader cognitive set, we excluded both PiB-PET and the sparse cognitive scores and used FreeSurfer^24^ to derive cortical ROI metrics (surface, thickness, volume) from the T1 scans. MIRIAD also contains 69 subjects (46 AD, 23 CN) with T1 MRI only; we extracted the same ROI metrics, but no PET or extensive cognitive data were available. These two datasets let us evaluate our framework’s generalization and robustness under different imaging scanners and modality gaps.

### Graph edges (Deep learning models)

#### Skull-stripping models

**Fig. 4:**
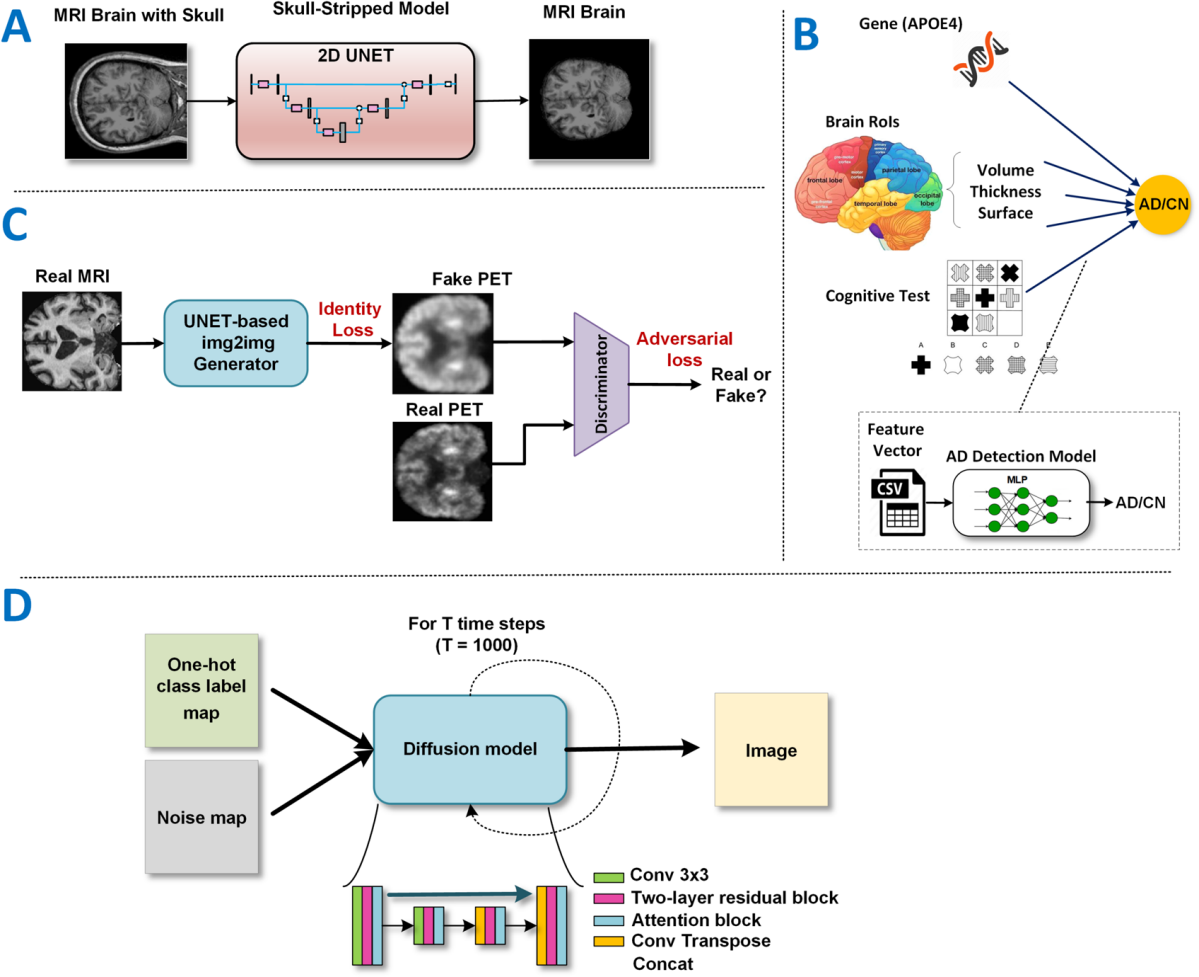
Architectural overview of models and components in our graph-based machine learning framework. (**A**) Skull Stripping - 2D-UNET Model. (**B**) Feature-based Detection - Multi-Layer Perceptron Model. (**C**) Image Translation - StyleGAN-based Generative Model. (**D**) Label Translation - Conditional Diffusion Model.

We employed a 2D-UNET model^31^ to perform skull-stripping on both MRI and PET images (Fig. 4A). Traditionally, UNET models generate a binary mask that is applied to the original MRI or PET image to isolate brain tissue. In our adaptation, we modified the final classification layer of the UNET model to directly output the skull-stripped image, bypassing the binary mask generation step. This modification made the model end-to-end differentiable, facilitating backward gradient flow throughout the entire network. We used the Mean Squared Error (MSE) as a loss function during training. For training, the MRI skull-stripping model was trained using the OASIS dataset, while the PET skull-stripping model was trained using the ADNI dataset. We used FreeSurfer to create skull-stripped images as ground truth for PET.

Although tools such as FreeSurfer, ANTs^32^, and SPM^33^ are widely used for brain extraction, a learned model offers four key advantages: (i) End-to-end integration within our graph allows potential joint fine-tuning with subsequent classification edges, (ii) faster inference is achieved by slice-by-slice prediction on a GPU, significantly reducing runtime compared to conventional pipelines, (iii) customization via domain-specific augmentations enables improved adaptation to heterogeneous AD imaging data, and (iv) modular & efficient workflow reduces external dependencies and ensures consistency in preprocessing across large-scale datasets. Overall, this deep-learning–based approach consolidates multiple stages of preprocessing, augmentation, and classification into a unified, flexible pipeline designed to enhance both efficiency and accuracy in Alzheimer’s Disease detection.

During training, we optimized all models with the Adam optimizer (initial learning rate=1e-4) and a ReduceLROnPlateau scheduler. These hyper-parameters were kept consistent across every edge in the graph.

#### Image-based AD detection model

In Paths 1 and 2 in Fig. 1, we implemented AD detection using T1 MRI and FluoroDeoxyGlucose (FDG) PET scans. MRI provides detailed anatomical images, showing structural changes such as neurodegeneration, while PET offers functional imaging, revealing metabolic deficits indicative of Alzheimer’s disease. Incorporating both MRI and PET detection models on our graph allows for a more comprehensive approach to AD detection by integrating structural and functional brain information.

In these paths, we used the 2D-ResNet18 model^34^ to classify MRI and PET scans as either AD or CN. ResNet18, known for its residual learning framework, is well-suited for this task due to its ability to learn complex patterns in brain images, such as structural abnormalities in MRI or metabolic changes in PET scans. By leveraging both imaging modalities, ResNet18 captures complementary anatomical and functional information, enhancing the accuracy of AD detection.

While the original MRI and PET scans used in this study were 3D, we extracted 2D slices from the coronal plane for processing. Each patient had a varying number of slices from the bottom to the top of the brain, so we applied interpolation to normalize all samples to a fixed set of 100 slices per subject. For training the PET and MRI classifiers, we used these individual slices. However, during the testing phase, we passed each of the 100 slices through the classifier individually to obtain a class score for every slice. The final classification score for each subject was then computed by averaging the class scores across all 100 slices, allowing for a more robust subject-level diagnosis.

#### Feature-based AD detection model

In Paths 3-7, depicted in Fig. 1, we implemented Alzheimer’s Disease (AD) detection using various features extracted from the ADNI dataset. These features encompass critical biomarkers associated with AD progression, including structural brain metrics such as cortical volume (Path 3), cortical thickness (Path 4), and surface area (Path 5) of key brain regions of interest (ROIs).These region-level measures are derived from ADNI’s standard FreeSurfer pipeline, which employs an anatomical parcellation (the Desikan–Killiany atlas^35^) to segment T1-weighted MRI scans into 68 cortical regions plus subcortical structures. Leveraging these precomputed metrics ensures consistency with widely used neuroimaging standards and facilitates comparisons across ADNI-based studies.

Additionally, we incorporated genetic information, specifically the presence of the APOE4 allele (Path 6), a known genetic risk factor for AD. Path 7 uses a comprehensive set of cognitive tests scores, including CDRSB, ADAS11, ADAS13, ADASQ4, MMSE, RAVLT-immediate, RAVLT-learning, and RAVLT-forgetting, which are standardized cognitive tests commonly used in clinical settings to evaluate AD patients. To analyze these diverse numerical features, we designed and trained specific Multi-Layer Perceptron (MLP) models, customizing the architecture of each MLP to match the dimensionality and characteristics of the corresponding feature set (Fig. 4B).

#### Image2Image generative model

In Paths 8 and 9, we used a StyleGAN-based image-to-image generative model^19^ for bidirectional image translation between MRI and PET scans, facilitating the generation of synthetic images across modalities. This generative model leverages a style injection mechanism, allowing the model to effectively transfer fine-grained style features from one modality to another. Our GAN training framework includes a PatchGAN discriminator^36^, which evaluates the authenticity of the generated images at the patch level rather than the full image during the training process. This approach allows for finer-grained discrimination, ensuring that the synthetic images accurately capture small, localized features critical for diagnosis (Figure 4C).

We optimize the generator using a combination of the three losses (pixelwise L1, perceptual^37^ and adversarial^38^ ). To further enhance the clinical validity of the generated images, we employed a composite function approach that combines image generation and image classification models (Eq. 2). Incorporating the classifier loss function ensures that synthetic images are realistic, clinically relevant, and accurately represent specific diagnostic classes. This approach involves our pre-trained image classifier to compute the cross-entropy loss on generated samples. This can be mathematically represented as: *C*(*G*(*c*, *z*)) where *c* is the input image, *G* is the image generator, transforming an image from one modality to another, and *C* is a pre-trained AD/CN classifier evaluating the generated image. For example, for the MRI2PET generator *G*, *c* represents the MRI scan, *G*(*c*, *z*) is the generated synthetic PET image, *C*(*G*(*c*, *z*)) yields the classifier predicted label, AD or CN.

2 $$\begin{aligned} \begin{aligned} {L}_G =&\ \lambda _{\text {L1}} \mathbb {E}_{\textbf{x}, \textbf{c} \sim p_{\text {data}}, \textbf{z} \sim p_{\textbf{z}}} [\Vert \textbf{x} - G(\textbf{c}, \textbf{z}) \Vert _1] \\&+ \lambda _{\text {VGG}} \mathbb {E}_{\textbf{x}, \textbf{c} \sim p_{\text {data}}, \textbf{z} \sim p_{\textbf{z}}} [\Vert \phi (\textbf{x}) - \phi (G(\textbf{c}, \textbf{z})) \Vert _1] \\&+ \lambda _{\text {adv}} \mathbb {E}_{\textbf{c} \sim p_{\text {data}}, \textbf{z} \sim p_{\textbf{z}}} [(D(\textbf{c}, G(\textbf{c}, \textbf{z})) - 1)^2] \\&+ \lambda _{\text {C}} \mathbb {E}_{ \textbf{c} \sim p_{\text {data}}, \textbf{z} \sim p_{\textbf{z}}} \left[ - \sum _{i} y_i \log (C(G(\textbf{c,z}))_i) \right] \end{aligned} \end{aligned}$$

where $\textbf{x}$ denotes a real target image and condition $\textbf{c}$ denotes the real input image from the data distribution $P_{data}$, $\textbf{z}$ is the noise input sampled from the noise distribution $P_{Z}(z)$ and G(c, z) is the image generated by the generator conditioned on the input image $\textbf{c}$ and the noise vector $\textbf{z}$, $\phi$ serves as the feature extractor (VGG network) and D represents the PatchGAN discriminator and $y_i$ in cross-entropy loss represents the true class label for class i.

In our loss functions, applying different weights ($\lambda$) helps to balance the multiple objectives of a model. Each part of the loss function has a specific role, and the weights ensure that the model focuses on the most important aspects of the task. We achieved the best results by using the following weights for each loss term: $\lambda _{\text {L1}} = 50$ , $\lambda _{\text {VGG}} = 100$, $\lambda _{\text {adv}} = 1$ and $\lambda _{\text {C}} = 50$.

#### Label2Image generative model

In Paths 10 and 11, we employed two diffusion models^39^ to generate synthetic MRI and PET images conditioned on the diagnostic status (AD or CN). To optimize the performance of these diffusion models, we modified their original architecture into a three-step encoding/decoding sequence. Each step integrates 3x3 convolutional/deconvolutional layers for efficient image processing, residual blocks to retain critical information and prevent vanishing gradient issues, and attention blocks to emphasize important image features (Fig. 4D).

The loss function for the Label2Image diffusion model (Eq. 3) is the Mean Squared Error loss to ensure the generated images retain their original structural integrity by matching the predicted noise to the original noise , conditioned on the label mask *y*. Besides, to further ensure the diagnostic relevance of the generated images, we applied the same composite function approach discussed in the Image2Image GAN model and we integrated the cross-entropy loss from our pre-trained AD/CN classifier into the diffusion model’s training process.

3 $$\begin{aligned} L_{\text {label2image}} = \mathbb {E}_{\textbf{x}_0, \mathbf {\epsilon }, \textbf{t}} \left[ \left\| \mathbf {\epsilon } - \mathbf {\epsilon }_\theta (\textbf{x}_t, t, \textbf{y}) \right\| ^2 \right] + \mathbb {E}_{ \mathbf {x_0}} \left[ - \sum _{i} y_i \log (C(G(\mathbf {x_0,y}))_i) \right] \end{aligned}$$

where $\textbf{x}_0$ is the original image, $\mathbf {\epsilon }$ is the true noise added to the image, $\textbf{x}_t$ is the noisy image at time step *t*, $\textbf{y}$ is the label mask matrix corresponding to $\textbf{x}_0$ (e.g., a matrix of all ones for label 1 and all zeros for label 0), $\mathbf {\epsilon }_\theta (\textbf{x}_t, t, \textbf{y})$ is the predicted noise by the neural network parameterized by $\theta$, given the noisy image $\textbf{x}_t$, and time step *t*, and the label mask $\textbf{y}$, $\mathbb {E}$ denotes the expectation over the data distribution, noise distribution, and time steps. In cross-entropy loss, *G* is the Label2Image diffusion model and *C* is our pre-trained AD/CN classifier evaluating the generated image and $y_i$ represents the true class label for class *i*.

#### Graph MLP output model

To integrate the outputs (AD/CN probability scores) from various brain feature classifiers, we chose to connect them to a Multi-Layer Perceptron (MLP) model rather than using traditional voting or ensemble methods. This approach allows the MLP to learn an optimal combination of the classifiers’ outputs. This MLP model consists of 14 inputs (7 modalities × 2 AD/CN scores = 14), four fully connected layers with 256, 128, 64, and 2 neurons, respectively, with ReLU activations applied after each layer and dropout layers (dropout-rate = 0.5) applied after the second and third layers. We used cross-entropy for the loss function. To make the MLP robust to missing data, we randomly mask 0–5 of the seven modality scores at every training step, exposing the network to a wide spectrum of incomplete-input scenarios.

### Computational cost and runtime

To provide a practical perspective on adopting our multi-modal framework, we measured both training and inference times for each deep-learning component. All experiments were carried out on an AWS p3.8xlarge instance equipped with 4 Ã— NVIDIA Tesla V100 (16 GB each), an Intel Xeon E5 2686 v4 CPU clocked at 2.30 GHz, and 64 GB of system RAM. Supplementary Table S2 summarizes these approximate runtimes, showing the computational resources needed to replicate our approach. Notably, once the initial training is completed, deployment (inference) is relatively quick, making it feasible for large-scale testing. The heaviest component (MRI $\leftrightarrow$ PET image-translation models) was trained for $\sim$150 h but infers a single subject in 246 ms. Lightweight MLP paths train in less than 1 min and predict in less than 0.2 ms.

## Supplementary Information

Supplementary Information.

## Data Availability

In our study, we employed four datasets for training and testing the edges of our graph: ADNI, OASIS-3, AIBL, and MIRAID datasets. The data from these datasets is not openly available but can be accessed upon request through their respective websites, subject to approval procedures. − The ADNI dataset uses as the primary resource for training all the models. Access to the ADNI dataset is available through its official homepage: (http://adni.loni.usc.edu). − The OASIS-3 MRI dataset was utilized for training the MRI skull stripping model. The OASIS-3 data can be requested via the OASIS Brains website (https://www.oasis-brains.org). − AIBL and MIRAID served as external datasets and were used for evaluating the framework’s performance. These datasets are available through their websites: AIBL: https://aibl.csiro.au/adni/index.html. MIRAID: https://www.ucl.ac.uk/drc/research-clinical-trials/minimal-interval-resonance-imaging-alzheimers-disease-miriad

## Code availability

The Python scripts implementing our developed methodologies are publicly available for download on our GitHub repository: https://github.com/NajmehMa/MADD.
