# Supplementary material for: A multi-modal graph-based framework for Alzheimer’s disease detection
Source: Sci Rep. 2025 Jul 2;15:22684. doi: 10.1038/s41598-025-05966-2 (PMC12218577; doi:10.1038/s41598-025-05966-2)
Supplement: Supplementary file 1 — Supplementary Information. [file 41598_2025_5966_MOESM1_ESM.pdf]

A Multi-Modal Graph-Based Framework for Alzheimer's Disease Detection

Najmeh Mashhadi[1] **and Razvan Marinescu**[2,*]

[1,2]Department of Computer Science and Engineering, University of California, Santa Cruz, Santa Cruz, CA, USA
[*]ramarine@ucsc.edu

Modality	Num of Subjects		Num of Scans	
	AD	CN	AD	CN
T1 MRI Scan	402	528	1121	2667
FDG PET Scan	307	347	555	941
Brain ROIs Surface	420	1100	-	-
Brain ROIs Volume	338	417	-	-
Brain ROIs Thickness	338	417	-	-
APOE4 Gene Expression	411	541	-	-
Cognitive Tests	411	541	-	-

Table S1. ADNI cohort composition. Counts of Alzheimer's disease (AD) and cognitively normal (CN) subjects and scans for each image-based and feature-based modality used in our study.

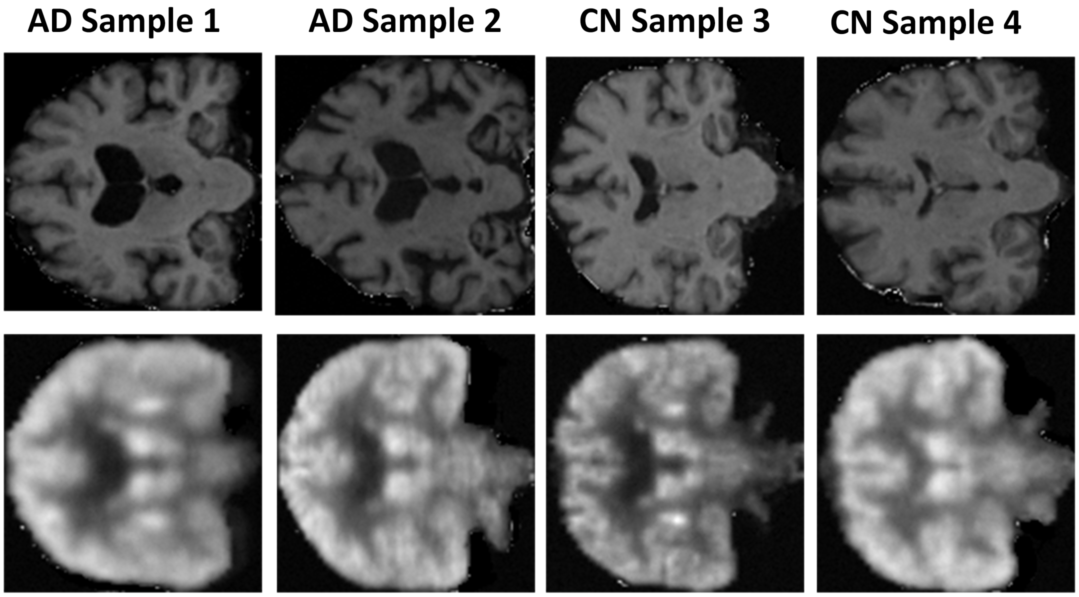

Figure S1. Synthetic MRI and PET examples. Four representative pairs of synthetic MRI and PET images generated by the label-to-image diffusion models.

Model	Training Time	Inference Time Per Subject	Dataset Size
MRI Skull-Stripping	$\sim$ 21 h	224 ms	461086 2D slices (batch size=128)
PET Skull-Stripping	$\sim$ 18 h	224 ms	337860 2D slices (batch size=128)
MRI-to-Label Model	< 1 h	211 ms	113640 2D slices (batch size=64)
PET-to-Label Model	< 1 h	211 ms	44880 2D slices (batch size=64)
ROIs-Volume-to-Label Model	< 1 min	< 0.2 ms	775 subjects (batch size=8)
ROIs-Thickness-to-Label Model	< 1 min	< 0.2 ms	775 subjects (batch size=8)
ROIs-Surface-to-Label Model	< 1 min	< 0.2 ms	1520 subjects (batch size=8)
APOE4-to-Label Model	< 1 min	< 0.2 ms	952 subjects (batch size=8))
Cognitive-Tests-to-Label Model	< 1 min	< 0.2 ms	952 subjects (batch size=8)
MRI-to-PET Model	$\sim$ 150 h	246 ms	36030 2D slices (batch size=128)
PET-to-MRI Model	$\sim$ 150 h	246 ms	36030 2D slices (batch size=128)
Label-to-MRI Model	$\sim$ 30 h	$\sim$ 3 s	3788 2D slices (batch size=64)
Label-to-PET Model	$\sim$ 30 h	$\sim$ 3 s	1496 2D slices (batch size=64)

Table S2. Model runtimes and dataset sizes. Summary of approximate training and inference times for each model component, along with the dataset size used in our experiments. All timings were measured on an AWS p3.8xlarge instance (4 × NVIDIA V100 16 GB).

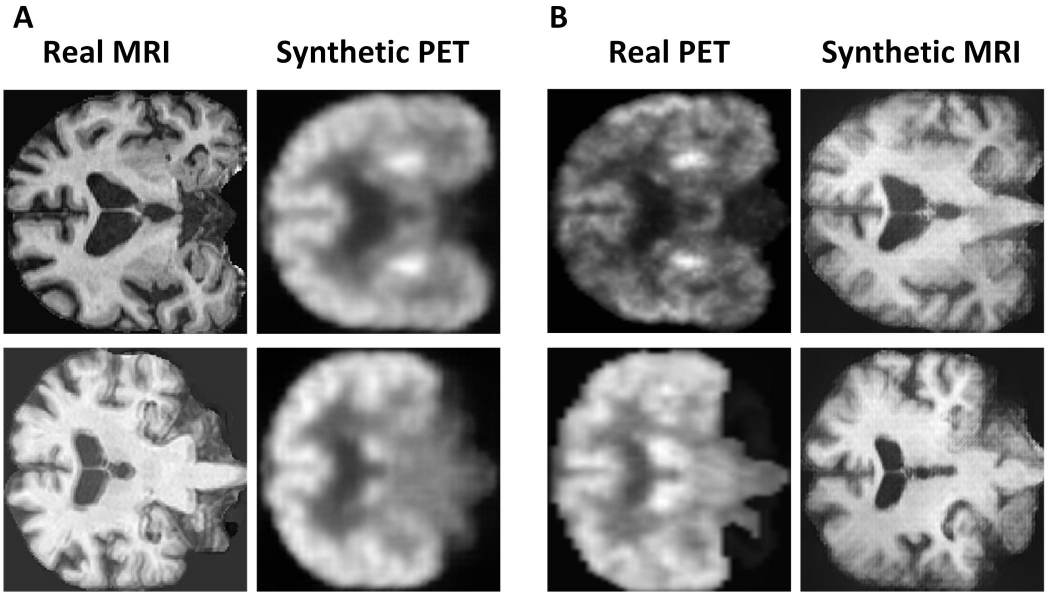

Figure S2. Bidirectional MRI–PET image-translation examples. (A) Two representative examples of synthetic PET images generated by the MRI-to-PET Image translation model. (B) Two representative examples of synthetic MRI images generated by the PET-to-MRI Image translation model.
